# Intramuscular Neural Distribution of the Serratus Anterior Muscle: Regarding Botulinum Neurotoxin Injection for Treating Myofascial Pain Syndrome

**DOI:** 10.3390/toxins14040271

**Published:** 2022-04-11

**Authors:** Kyu-Ho Yi, Ji-Hyun Lee, Hee-Jin Kim

**Affiliations:** 1 COVID-19 Division, Wonju City Public Health Center, Wonju-si 26417, Korea; kyuho90@daum.net; 2 Division in Anatomy and Developmental Biology, Department of Oral Biology, Human Identification Research Institute, BK21 FOUR Project, College of Dentistry, Yonsei University, Seoul 03722, Korea; jhlee119@yuhs.ac; 3 Department of Materials Science & Engineering, College of Engineering, Yonsei University, Seoul 03722, Korea

**Keywords:** myofascial pain syndrome, Sihler’s method, serratus anterior, trigger point injection

## Abstract

The serratus anterior muscle is commonly involved in myofascial pain syndrome and is treated with many different injective methods. Currently, there is no definite injection point for the muscle. This study provides a suggestion for injection points for the serratus anterior muscle considering the intramuscular neural distribution using the whole-mount staining method. A modified Sihler method was applied to the serratus anterior muscles (15 specimens). The intramuscular arborization areas were identified in terms of the anterior (100%), middle (50%), and posterior axillary line (0%), and from the first to the ninth ribs. The intramuscular neural distribution for the serratus anterior muscle had the largest arborization patterns in the fifth to the ninth rib portion of between 50% and 70%, and the first to the fourth rib portion had between 20% and 40%. These intramuscular neural distribution-based injection sites are in relation to the external anatomical line for the frequently injected muscles to facilitate the efficiency of botulinum neurotoxin injections. Lastly, the intramuscular neural distribution of serratus anterior muscle should be considered in order to practice more accurately without the harmful side effects of trigger-point injections and botulinum neurotoxin injections.

## 1. Introduction

Myofascial pain syndrome (MPS) is extremely common, occurring in up to 95% of individuals [1]. Repetitive movements and incorrect posturing habits contribute to the advancement of MPS by triggering overload on a particular muscle; the serratus anterior (SA) muscle is the most commonly involved [2]. As a part of MPS, serratus anterior myofascial pain syndrome (SAMPS) is separately named due to its frequency [2]. Points with taut banded parts and pinched tenderness of the muscle belly are termed myofascial trigger points (MTrPs). SAMPS occurs with deep respiratory distress while running, repetitive coughing due to respiratory disease, lifting heavy loads, and other psychological stresses [3].

The cause of SAMPS is hyperactivated SA muscle contractions [4,5,6]. Pathological findings indicate an increase in the release of acetylcholine by the neuromuscular junction under relaxing conditions. Elevated and prolonged acetylcholine release generates persistent depolarization of the muscle fiber, which causes sarcomere shortening and involuntary muscle contraction [2]. This point is anatomically known to be the thickest muscle belly, with the most intramuscular neural arborization [7,8,9,10,11].

The therapeutic options for MPS include releasing MTrPs using injective agents such as botulinum neurotoxin (BoNT), lidocaine, steroids, normal saline, and combinations of agents. BoNT blocks neural transmission by stalling the release of acetylcholine at the neuromuscular junction and impedes muscle contraction [12]. In myofascial pain control, BoNT injection is renowned for offering better consequences than oral medications in terms of pain management and functional movement [13,14,15]. Therefore, injection of BoNT is widely used as a treatment option for MPS, especially SAMPS [16,17,18,19,20,21].

At present, BoNT injection is acknowledged as the most secure and effective treatment for inactivating the muscle [22,23,24,25]. The consequences of BoNT depend on uptake by the presynaptic membranes at the neuromuscular junction; thus, injections should be directed into the neuromuscular junction area where most neuromuscular junctions exist [12,26,27]. The significance of utilizing neuromuscular arborization-directed BoNT injections has been verified by clinical trials in the iliopsoas and bicep brachii muscles. These injections resulted in higher pain reduction as well as volume reduction compared to conventional injections [26,27].

However, intramuscular neural distribution of the muscle for accurate injection points is necessary for BoNT, as excessive amounts of BoNT may potentially cause the toxin to spread to the neighboring muscles, resulting in paralysis [28,29]. The adverse effect of paralyzed muscle is reported in cases of overdose of BoNT [30,31,32]. Moreover, repetitive and overdose of BoNT injections build up antibodies that will result in an insufficient treatment effect [30,31,33,34]. Consequently, BoNT should be injected into the arborized regions to enhance efficacy and decrease adverse effects. To direct the injection points while preventing these adverse effects, numerous studies have revealed the intramuscular neural arborization of various muscles, but not the SA [14,33,34,35,36,37,38,39,40,41,42,43]. This study aimed to reveal the intramuscular neural arborization of the SA and provide anatomical information of the SA muscle.

## 2. Results

### 2.1. Running of the Thoracic Nerve Trunk

The long thoracic nerve runs superficial to the SA muscle and pierces the muscle at each level until the seventh rib. Thirteen of the fifteen specimens had a trunk of the long thoracic nerve running at 30 to 50% throughout the level of the first to the seventh rib. The other two had the long thoracic nerve running down at 40 to 50% at the level of the first to the fourth rib and 30 to 40% at the level of the fifth to the seventh rib. 

### 2.2. Intramuscular Arborization Patterns of the SA Muscle

**Figure 1:**
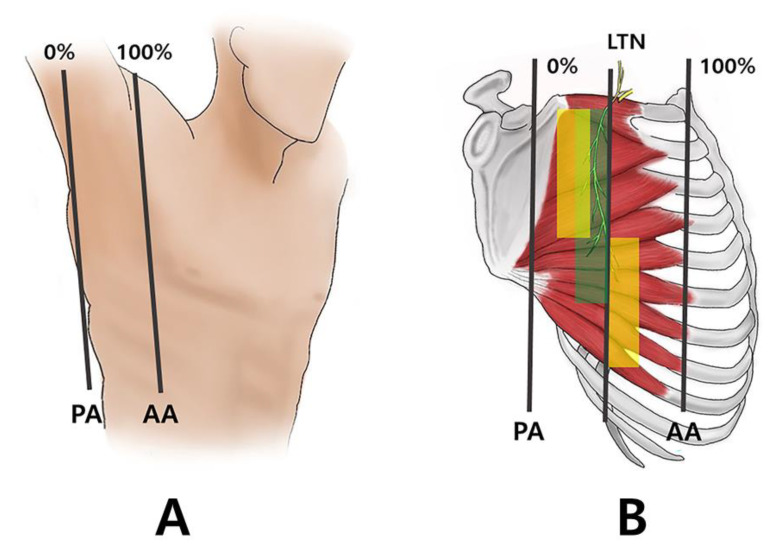
Specimens were harvested according to two anatomical lines: the anterior axillary line (AA) and posterior axillary line (PA). The AA was 100% to PA of 0%, respectively (**A**). The long thoracic nerve runs superficial to the SA muscle and pierces into the muscle at each level until the 7th rib. The specimens had a trunk of the long thoracic nerve running on 30 to 40% throughout the level of 1st to 7th rib (green shaded). The intramuscular arborization patterns were the largest: the 6th to 9th rib portion had between 50% to 70% and the 1st to 5th rib portion had between 20 to 40%. The injection should be guided to these arborized areas (**B**).

Twelve of the fifteen SA muscles had two regions in which the arborization patterns were the largest: the sixth to the ninth rib portion had between 50 and 70% and the first to the fifth rib portion had between 20 and 40%, following three anatomical lines: the anterior (100%), middle (50%), and posterior axillary line (0%) (Figure 1B). The other two had the largest patterns in the fourth to the ninth rib portion, between 50% and 60%; the first to the third rib portion had between 20% and 30%. The last muscle had the largest patterns in the fourth to the ninth rib portion, between 50% and 70%; the first to the third rib portion had between 30 to 40%.

## 3. Discussion

### 3.1. Anatomy of the SA Muscle

The SA muscle is a flat and wide muscle covering the lateral ribs; it is anatomically divided into three muscle bellies [2]. It consists of an upper, middle, and lower muscle belly, each of which contribute to the movement of the scapular bone during upper extremity actions [44]. The upper belly of the SA lies parallel to the first rib and inserts into the superior angle of the scapula [45]. The middle belly of the SA originates from the second, third, and fourth ribs and inserts into the medial scapular border. The lower muscle belly of the SA is where the MTrPs frequently exist, originating from the fifth to the ninth ribs and inserting into the inferior angle of the scapula [45,46]. The SA muscle is innervated by the long thoracic nerve, which originates from the anterior rami of spinal nerves C5–C7 [47,48]. The long thoracic nerve runs superficially over the SA muscle along the anterior axillary line. The SA muscle is mostly involved in upper extremity movements; however, it is the prime stabilizer of the shoulder girdle and acts on shoulder flexion, abduction, and upward rotation [44].

### 3.2. MPS in SA Muscle

MPS is a chronic pain disorder caused by MTrPs situated at the muscle belly; it has been recognized as the main cause of pain in 85% of patients attending pain clinics [48,49]. SA muscle MTrPs may be triggered by muscle strain during excessive running, overloaded weightlifting, or repetitive coughing, especially susceptible to torsional stresses. Another cause of MTrPs initiation in the SA muscle is breast surgery due to cancer or esthetic purposes [50].

Studies have revealed that sarcomere shortening is related to MTrP etiology, and the shortening is due to an increase in activation of the neuromuscular junction and its over-release of acetylcholine. In addition, a large quantity of calcium released at the sarcoplasmic reticulum over a dysfunctional ryanidine receptor causes prolonged muscle contraction [51]. Therefore, to release muscle contraction, BoNT is currently frequently used as an injective agent for MPS [51,52,53]. The primary known therapeutic effects are releasing muscular contractions and alleviating the vicious pain cycle [54,55,56]. Injection treatment of MPS with local anesthetics is reported to be highly effective and currently represents the gold standard [57]. The local anesthetics are thought to bring relief from muscle tightness. Additionally, the injection of BoNT is another treatment option inhibiting the diffusion of neurotransmitters in the peripheral nerve, avoiding peripheral sensitization [58,59].

In the study of Kamanli et al., lidocaine injection is more practical, since it causes less disturbance than dry needling and is more cost effective than BoNT injection, and it seems to be the treatment of choice in MPS [60]. However, they have proposed that BoNT could be selectively used in MPS patients resistant to conventional treatments. In many of the assessment scores with lidocaine, dry needling and BoNT injection, depression and anxiety scores significantly improved only in the BoNT-injected group [60].

Neuromuscular junctions are the underlying causes of MPS; therefore, injecting BoNT and other injective treatments such as lidocaine, steroids, and normal saline are frequently performed to target the neuromuscular junctions [61,62,63]. Unlike oral medications and lidocaine injections that have short-term effects, the effectiveness of BoNT treatment in MPS has been known to continue for up to 4 months [62,63].

As BoNT acts on the neuromuscular junction, accurate anatomical knowledge of the neuromuscular arborization patterns of the SA muscles is vital for achieving the highest relief with the smallest possible dose of BoNT. Although BoNT procedures are minimally invasive compared to surgical intervention, there is a probability of damaging the nerve trunks that are not present near the neural arborized area. Therefore, precise knowledge of the anatomical features of the SA muscle should be considered. In this study, we carried out Sihler’s staining, which is a whole-mount staining procedure that stains myelin sheaths and is effective in tracing the nerve endings without destroying the nerves [14,33,34,35,64]. The application of Sihler’s staining to the SA muscle will enable an accurate and thorough understanding of the neural distribution.

Moreover, identifying the neural arborization area of the SA muscle is important in diagnosing long thoracic nerve palsy [65]. Surface electromyography in the SA muscle is challenging because multiple thin digitations make it difficult to place the electrode for recording [66,67]. When detecting long thoracic nerve palsy, the technical limitations of electromyography are interrupted signals from the neighboring muscles and difficulty with accurate electrode placement since the SA is not a bulky muscle.

At present, there is no anatomical guidance for the injection or EMG of the SA muscle. The authors acknowledge the following limitations in the current study. The results are solely based on the analysis from Sihler staining of cadavers’ SA muscles. Additionally, the cadavers are from elderly people with an average age of 76.6, and from a single race (Korean).

In this study, we have revealed intramuscular distribution of the SA muscle that might help clinicians guiding electromyography and injective treatments including BoNT, lidocaine, normal saline, and steroids. In the anatomical aspect, clinicians should be able to carefully target the three regions in the middle portion, between the sixth to ninth rib portion and the first to the fifth rib (Figure 1B).

## 4. Materials and Methods

This study was performed in accordance with the principles outlined in the Declaration of Helsinki. Informed consent and approval were obtained from the families of the cadavers before the dissections were performed and approved by the Institutional Review Board of Yonsei University College of Medicine (approval number 20-006, approved date: 26 February 2020). A total of 15 SA muscles from Korean cadavers (5 men and 4 women with a mean age of 76.6 years; range, 73–95 years) were dissected in Yonsei University medical center from May 2020 to October 2020, and modified Sihler staining was applied to clarify the intramuscular neural arborization patterns.

**Figure 2:**
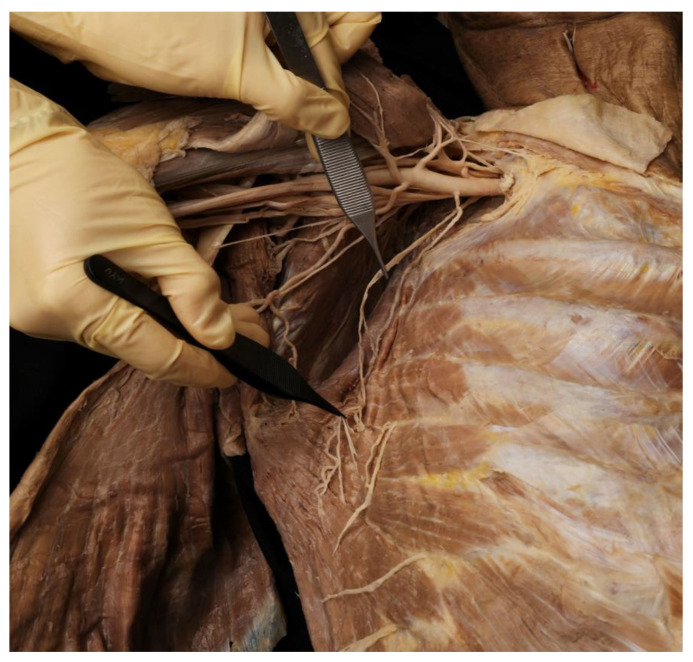
The serratus anterior muscle the long thoracic nerve running over the muscle. The long thoracic nerve has been pointed out by the forceps.

Before dissection, the SA muscles were aligned in their anatomical positions (Figure 2). The arborizing patterns of the SA muscles were tracked according to the three anatomical lines: anterior (100%), middle (50%), posterior axillary line (0%), and from the first to the ninth ribs (Figure 1A).

**Figure 3:**
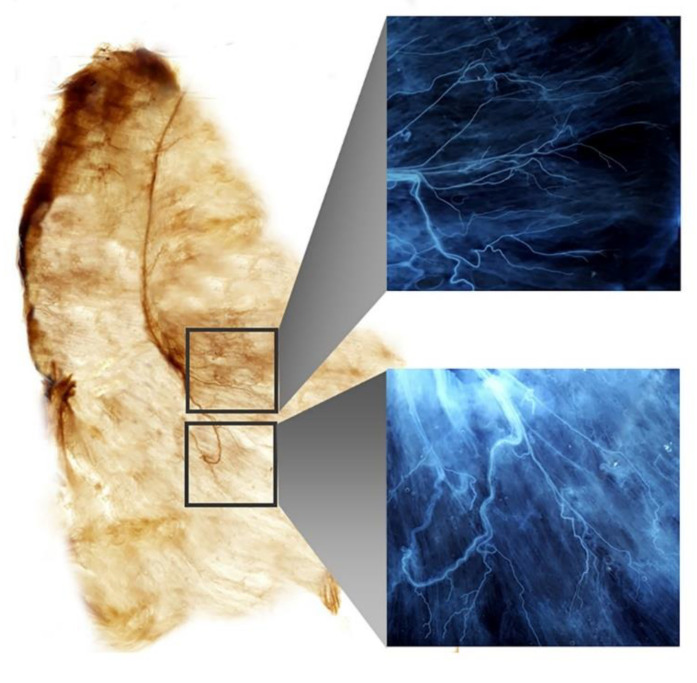
The result of Sihler’s staining of the serratus anterior muscle. The intramuscular neural distribution of the serratus anterior muscle is observed with enlarged views.

The SA muscles underwent Sihler staining, as modified by Liem and Douwe van Willingen (Figure 3) [67].

**Figure 4:**
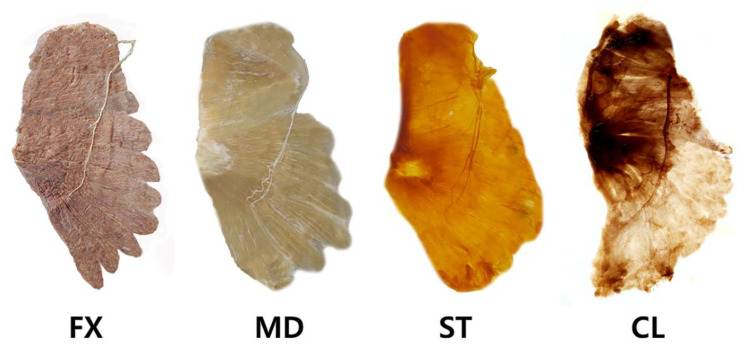
The serratus anterior muscle underwent modified Sihler’s method. The method consists of stages of fixation (FX), maceration and depigmentation (MD), decalcification, staining (ST), and clearing (CL).

This technique involves several steps to acquire the visual representation of the intramuscular neural arborization pattern. The changes over Sihler’s method of the SA specimens are shown in Figure 4.

Following Sihler staining, the SA muscles were divided into 10 sections according to the vertical lines from the anterior and posterior axillary lines and the curved lines of the first to ninth ribs.

### Modified Sihler Staining

Fixation: The SA muscles were stored for one month in a container filled with 10% un-neutralized formalin. The solution was replaced with fresh solution whenever it turned cloudy.

Maceration and depigmentation: The fixed SA specimens were washed in running water for an hour. Then, they were placed for one month in a container filled with 3% aqueous potassium hydroxide and hydrogen peroxide solution.

Decalcification: The depigmented SA specimens were then placed in Sihler I solution, a compound of glycerin, glacial acetic acid, and aqueous chloral hydrate.

Staining: The decalcified SA specimens were then stained with the Sihler II solution, a compound of glycerin, aqueous chloral hydrate, and acetic acid. The staining process takes 30–35 days for intramuscular nerve visualization.

De-staining: The stained SA specimens were cleansed in a container filled with Sihler I solution. This step is used to de-stain the SA muscle fibers so that only the intramuscular nerve distributions are visualized.

Neutralization: The de-stained SA specimens were neutralized in clean water for half an hour. Consequently, the SA specimens were placed in a solution of 0.05% lithium carbonate.

Clearing: Finally, the neutralized SA specimens were taken into the clearing stage with glycerin by increasing the concentrations from 20% to 100%. This stage took nearly 4–5 h.

## Data Availability

Not applicable.
